# Public-Private Partnerships With Unhealthy Commodity Industries: Are They Undermining Real Progress in Non-communicable Disease Prevention? Comment on "Competing Frames in Global Health Governance: An Analysis of Stakeholder Influence on the Political Declaration on Non-communicable Diseases"

**DOI:** 10.34172/ijhpm.2021.118

**Published:** 2021-08-30

**Authors:** Chiara Rinaldi

**Affiliations:** Department of Health Services Research and Policy, London School of Hygiene and Tropical Medicine, London, UK.

**Keywords:** Public-Private Partnerships, Non-Communicable Diseases, Global Health Governance, Conflicts of Interest

## Abstract

Public-private partnerships (PPPs) and whole-of-society approaches are increasingly common in public health promotion and non-communicable disease (NCD) prevention, despite a lack of evidence in favour of their effectiveness in improving health outcomes. While PPPs may have advantages, they also give industry actors more influence over the design and implementation of public health strategies and interventions. Partnering with unhealthy commodity industries in particular – including the alcohol and ultra-processed food and beverages industries – can pose significant risks to public health due to these industries’ deep-rooted conflicts of interest. In this commentary, I reiterate Suzuki and colleagues’ message about the importance of assessing and managing conflicts of interest before engaging with non-state actors through PPPs or other forms of engagement.

## Background

 The commercial determinants of health, or “factors that influence health which stem from the profit motive” (p. 687),^1^ are a growing area of interest in public health research. The impact of unhealthy commodity industries – including the tobacco, alcohol and ultra-processed food and beverages industries – on non-communicable diseases (NCDs) is of particular concern due to the ways in which those actors have been able to influence policy-making processes. Unhealthy commodity industries have a clear conflict of interest in NCD prevention because of their financial interests in producing, marketing and selling products that contribute to the burden of NCDs.^3^ However, while the conflicts of interest of the tobacco industry are generally well understood and managed, Suzuki and colleagues’ analysis highlights the need to identify and evaluate other industries that have significant and often irreconcilable conflicts of interest in the prevention of NCDs.^2^

 In their analysis of stakeholder influence on the United Nations (UN) Political Declaration on NCDs, Suzuki et al document the extent to which the Declaration reflects the policy positions advocated by different public, private and third sector stakeholders during the consultation process.^2^ They identified divergent framing between stakeholder groups, and inequalities in whose framing was adopted by the UN in the final Declaration. Notably, the issues raised by non-governmental organizations (NGOs) and low- and middle-income countries (LMICs) were less likely to be adopted than those raised by industry stakeholders and high-income countries. As Suzuki and colleagues’ analysis shows, unhealthy commodity industries like the alcohol and ultra-processed food and beverages industries are often treated as ‘partners’ in NCD governance despite the growing evidence that the framing and strategies they adopt interfere with the implementation of evidence-based public health measures.^2^ In line with previous analyses, the authors question whether and how unhealthy commodity industries should be formally involved in global NCD policy deliberations.

## Public-Private Partnerships and Whole-of-Society Approaches

 One of the main themes that emerged from Suzuki and colleagues’ analysis is the promotion of public-private partnerships (PPPs) and ‘whole-of-society’ approaches, predominantly by private sector actors. In recent years, PPPs have increasingly been adopted as a tool to address common risk factors for the development of NCDs – such as unhealthy diets, physical inactivity and obesity – and other health-related challenges.^4^ PPPs represent a shift in governance for health, in which responsibility is shared between public bodies and other societal stakeholders.^5^ ‘Multi-stakeholder’ or ‘whole-of-society’ approaches are promoted by the UN, for example through Sustainable Development Goal 17 which includes the target to “encourage and promote effective public, public-private and civil society partnerships.”^6^ While ‘whole-of-society’ approaches are broader than PPPs and often aim to engage civil society stakeholders, there is evidence that the term is used by representatives of the alcohol and food industry to justify and promote industry involvement in policymaking, and to oppose attempts to limit involvement due to conflicts of interest.^7,8^ The use of the terms ‘whole-of-society’ and ‘whole-of-government’ together without explicit differentiation, as seen in the UN Declaration on NCDs, further adds to this ambiguity about the meaning of different terms that indicate the engagement of sectors and stakeholders beyond health.^2^

## Effectiveness of PPPs in Preventing NCDs

 PPPs are often conceptualised as political practices through which corporations can contribute to addressing the negative externalities of their products or practices, while at the same time promoting their preferred regulatory environment by participating in decision-making processes.^9^ Both creating a more favourable image for themselves – often as part of corporate social responsibility – and having increased influence over strategies and interventions to address NCDs can be a reason for unhealthy commodity industries to partner with governments or international organisations, and to promote such partnerships in consultation processes. Benefits of PPPs for the public sector can include increased access to (financial) resources and expertise.

 However, there is no clear evidence in favour of the effectiveness of PPPs in public health promotion to date.^3,10,11^ A recent systematic review found that partnerships addressing NCD risk factors, which have high potential for conflicts of interest, are more likely to be reported as unsuccessful in reaching their goals compared to PPPs in communicable disease control.^10^ Moreover, the authors of the review expressed concern about the amount of ‘non-independent’ or industry-funded studies on PPPs, which were in turn more likely to be supportive of the evaluated partnerships. An evaluation of the UK Public Health Responsibility Deal, a PPP based on voluntary pledges from the alcohol and food and beverage industries, suggests that beyond just being ineffective, taking a PPP approach could delay the implementation of evidence-based public health regulation that is less favourable from a business perspective (eg, restrictions to the availability of products and fiscal policies).^11^

## The Promotion of Industry Framing of NCD Prevention Through PPPs

 As could be expected, Suzuki and colleagues’ analysis shows that industry stakeholders are opposed to regulation that could directly harm their business (eg, taxation on sugar-sweetened beverages).^2^ On the other hand, they note that “there was consensus from all constituencies including the alcohol industry to reduce the ‘harmful use of alcohol’ and eliminate ‘the marketing, advertising and sale of alcoholic products to minors’” (p. 9).^2^ While this appears positive, it does require comment. The alcohol industry tends to disproportionally focus on the harms of their products on the heaviest drinkers or particularly vulnerable groups (eg, minors) while rejecting harms in the wider population,^8,12^ despite evidence that the harms of alcohol start from a low level of consumption.^13^ This framing is also commonly used by the tobacco and ultra-processed food and beverage industries, and explains why their commitments and pledges have a similar, narrow focus.^3^ Limited support for interventions targeted at specific populations does not represent a comprehensive public health approach to NCD prevention – particularly given that the interventions that are favoured by unhealthy commodity industries (eg, education and awareness raising) tend to have a weaker evidence base.^14^ The increased influence of industry stakeholders in PPPs can lead to the adoption of a narrow individual-oriented framing of NCD prevention at the expense of an approach guided by public health values and evidence.^11^

 What is interesting to note about Suzuki and colleagues’ findings is that when there was no direct threat to their own business, industry stakeholder did not necessarily oppose more wide-ranging regulatory interventions.^2^ For example, an association of pharmaceutical industries supported the taxation of sugar-sweetened beverages. This further highlights the importance of carefully assessing and managing conflicts of interest when engaging in PPPs and acknowledging that some industry stakeholders are not in the position to contribute to decision-making process in a way that promotes evidence-based public health. It is therefore concerning that the UN Declaration on NCDs does not make any attempt to distinguish between the roles of different non-state actors and their (conflicting) interests despite thirty statements of support in the consultation process. In Suzuki and colleagues’ words, this could indeed be “the worst possible combination (promotion of PPPs without the management of conflict of interest) from the perspective of many NGOs and public health advocates” (p. 10).^2^

## Conclusion

 Given the current evidence base, the increasing shift towards PPPs could represent a concern for public health, specifically when it comes to the prevention and control of NCDs. The promotion of ‘multi-stakeholder’ and ‘whole-of-society’ approaches by the UN, with strong backing of unhealthy commodity industries, should therefore be done with caution. It is important to note that most evidence on PPPs focuses on formal partnerships established for the implementation of specific public health interventions with a shared goal. However, there are different ways in which partnerships can be organised, including more informal participation-based approaches with ‘engagement’ or ‘dialogue’ between sectors. Evidence on such partnerships remains limited. Regardless, we also need to carefully consider these other, less overt ways in which unhealthy commodity industries can influence strategies and approaches to NCD prevention, including through financial donations and through their status as ‘partners’ in policy deliberations and consultations. The World Health Organization Foundation has recently sparked controversy for accepting donations from harmful commodity industries, seemingly undermining Framework of Engagement with Non-State Actors principles which aim to protect WHO’s work from conflicts of interest.^15^ Suzuki and colleagues’ findings show that in the consultation process on the UN Declaration on NCDs, contestation was associated with a smaller likelihood of an issue being addressed and included in the Declaration with clear language. In this light, involving those with strong vested interests in policy outcomes in deliberations risks overshadowing important issues raised by NGOs and LMICs, giving industry stakeholders and high-income countries with strong ties to those industries disproportionate power. This is particularly concerning given the high and rising burden of NCDs in LMICs. Reconsidering the role that is given to stakeholders with significant conflicts of interest is an important opportunity to rebalance the power distribution in NCD governance and promote a greater role for civil society and people living with NCDs, who deserve to be protected by public health policies.

## Ethical issues

 Not applicable.

## Competing interests

 Author declares that she has no competing interests.

## Author’s contribution

 CR is the single author of the paper.

## Funding

 No funding was received for this commentary. CR is supported by a National Institute for Health Research (NIHR) School for Public Health Research (SPHR) Pre-doctoral Fellowship.
